# FAMILY MATTERS: EFFECTS OF UNFAIRNESS AND DISAGREEMENT ON DEMENTIA CAREGIVERS’ GAINS AND BURDEN

**DOI:** 10.1093/geroni/igae098.4190

**Published:** 2024-12-31

**Authors:** Suyoung Nah, Jyoti Savla

**Affiliations:** Virginia Tech, Blacksburg, Virginia, United States; Virginia Tech, Blacksburg, Virginia, United States

## Abstract

Although caregiving occurs within a family, the influence of family contexts on caregiver outcomes remains understudied. Building on the distributive justice framework and stress process theory, we examined whether an unequal division of care among adult siblings, perceived unfairness in the division, and family disagreement over parental care are associated with caregivers’ gains and role overload. We further explored whether perceived unfairness and family disagreement interact in predicting caregiver outcomes. Using data from the 2015 and 2017 National Health and Aging Trends Study and National Study of Caregiving, we examined adult child caregivers of persons living with dementia (N = 364 for cross-sectional analysis and N = 173 for longitudinal analysis; Mage = 54.0; White = 51%). Multivariate multilevel analysis was conducted to investigate the effects of these family contexts on caregivers’ gains and role overload. Caregivers who felt they were providing more care than their fair share experienced higher overload at initial assessment (β = 0.20, p =.001) and at follow-up (β = 0.23, p =.04). Greater family disagreement was associated with higher role overload at initial assessment (β = 0.33, p <.001). Family disagreement moderated the effect of perceived unfairness, such that the perception of providing less care was linked to lower gains at follow-up only when family disagreement was high (β = -0.67, p <.001). Findings highlight the importance of perceived unfairness and family disagreement in caregiver outcomes. Interventions should promote shared caregiving responsibilities and facilitate open communication within families about care dynamics.

